# Complete Blood Cell Count Parameters Predict Mortality in Patients with Hypersensitivity Pneumonitis

**DOI:** 10.3390/diagnostics15081038

**Published:** 2025-04-18

**Authors:** Matthaios Katsaras, Vasilina Sotiropoulou, Effrosyni Manali, Evangelia Fouka, Despoina Papakosta, Elisabeth Bendstrup, Lykourgos Kolilekas, Ioannis Tomos, Vasilios Tzilas, Paschalis Ntolios, Paschalis Steiropoulos, Ilias Papanikolaou, Athena Gogali, Konstantinos Kostikas, Panagiota Tsiri, Ourania Papaioannou, Elli Malakounidou, Eva Theohari, Ioannis Christopoulos, Fotios Sampsonas, Spyridon A. Papiris, Nikoletta Rovina, Demosthenes Bouros, Argyrios Tzouvelekis

**Affiliations:** 1 Department of Respiratory Medicine, University Hospital of Patras, 26500 Patras, Greece; matthewkat1@gmail.com (M.K.); atzouvelekis@upatras.gr (A.T.); 2 1st Respiratory Department, Sotiria Chest Hospital, Medical School, National and Kapodistrian University of Athens, 11527 Athens, Greece; 3 2nd Pulmonary Department, General University Hospital “Attikon”, Medical School, National and Kapodistrian University of Athens, 12462 Athens, Greece; 4 Pulmonary Department, G. Papanikolaou Hospital, Aristotle University of Thessaloniki, 57010 Thessaloniki, Greece; 5 Center for Rare Lung Diseases, Department of Respiratory Diseases and Allergy, Aarhus University Hospital, 8200 Aarhus, Denmark; 6 7th Pulmonary Department, Athens Chest Hospital “Sotiria”, 11527 Athens, Greece; 7 5th Pulmonary Department, Athens Chest Hospital “Sotiria”, 11527 Athens, Greece; 8 Department of Respiratory Medicine, Medical School, University General Hospital Dragana, Democritus University of Thrace, 68100 Alexandroupolis, Greece; 9 Pulmonary Department, Corfu General Hospital, 49100 Kontokali, Greece; 10 Department of Respiratory Medicine, Medical School, University of Ioannina, 45110 Ioannina, Greece

**Keywords:** hypersensitivity pneumonitis, biomarkers, monocyte count, eosinophil count

## Abstract

**Background:** Hypersensitivity pneumonitis (HP) represents a chronic lung disease with an unpredictable clinical course. There is a pressing need for clinically applicable prognostic biomarkers in patients with HP. **Methods:** This was an observational, retrospective study. We investigated the prognostic potential of complete blood count parameters in treatment-naïve patients diagnosed with HP between 15 December 2010 and 1 October 2023. Receiver operating characteristic (ROC) curve analysis identified the optimal cut-off thresholds for each parameter in terms of mortality prediction. **Results:** We included 129 patients diagnosed with HP [median age: 68.0 years (95% CI: 65.0 to 69.0), fibrotic HP: *n* = 85, 65.9%]. Patients with HP and an eosinophil count > 160 cells/μL [ROC curve, area under curve (AUC): 0.61] exhibited increased mortality risk compared to patients with HP and an eosinophil count ≤ 160 cells/μL [Kaplan–Meier, HR: 2.95 (95% CI: 1.36 to 6.42), *p* = 0.006]. Patients with HP and a monocyte count > 350 cells/μL (ROC curve, AUC: 0.52) had worse survival compared to patients with HP and a monocyte count lower than this threshold [Kaplan–Meier, HR: 2.48 (95% CI: 1.03 to 5.09), *p* = 0.04]. Patients with HP and an eosinophil–lymphocyte ratio (ELR) > 0.09 (ROC curve, AUC: 0.64) had a higher risk of mortality compared to patients with HP and ELR ≤ 0.09 [Kaplan–Meier, HR: 2.75 (95% CI: 1.3 to 5.78), *p* = 0.008]. **Conclusions:** This study demonstrated that eosinophil count, monocyte count, and ELR could be prognostic biomarkers in patients with HP. Further studies aiming to validate the prognostic potential of complete blood count parameters in patients with HP are greatly anticipated.

## 1. Introduction

Hypersensitivity pneumonitis (HP) is an inflammatory disease caused by inhalational exposure to organic antigens that has a heterogeneous and complex clinical presentation and natural history [1,2]. The diagnosis of HP has been frequently overlooked, considering that the disease is quite often misdiagnosed as idiopathic pulmonary fibrosis (IPF) or even stays undiagnosed. Indeed, patients with fibrotic HP quite often present with similar radiologic patterns and clinical behavior to IPF, and thorough investigation has revealed no identifiable inciting antigens [3].

HP encompasses a wide clinical spectrum—from reversible inflammation to irreversible fibrosis—and is influenced by environmental factors, host immunity, and disease chronicity. This heterogeneity complicates prognosis, making it crucial to identify markers that reflect underlying disease activity or progression risk [4,5]. At present, there is a major lack of knowledge on predictors of clinical outcomes that will guide timely and personalized therapeutic decisions in patients with HP. With a gradually increasing prevalence, the prognosis of HP’s clinical course still represents a major bottleneck for chest physicians and researchers [5]. Given that systemic inflammation may parallel disease course, peripheral blood markers such as complete blood count (CBC) components represent a readily available and non-invasive source of prognostic information.

Recent compelling evidence supports the role of CBC as a reliable prognosticator in patients with IPF. Increased monocyte counts have consistently predicted clinical outcomes in patients with IPF in multiple independent cohorts [6,7]. Given their encouraging results in predicting clinical outcomes in patients with IPF, an investigation into the prognostic usefulness of CBC parameters for patients with HP might hold promise; however, their clinical applicability in HP remains largely unexamined.

Of note, approximately 20–40% of HP individuals display non-specific airway hyperreactivity, while some patients exhibit acute flares of bronchoconstriction, peripheral blood eosinophilia, eosinophils in the bronchoalveolar lavage (BAL), elevated IgE levels, and a positive bronchodilation test. Environmental triggers, chronic immune-mediated inflammation, and prominent peribronchiolar involvement are key features of HP [8,9,10,11,12]. These can explain why HP has been characterized as the “asthma” of interstitial lung diseases (ILDs). Based on the above, an investigation into the prognostic potential of eosinophils in HP seems rational and may also reveal potential therapeutic targets. This study aimed to investigate the predictive accuracy of peripheral blood cell parameters in patients with HP.

## 2. Materials and Methods

This was an observational, retrospective multicentric study. Consecutive, treatment-naïve patients that had been diagnosed with HP between 15 December 2010 and 1 October 2023 were retrospectively included in the study.

We included patients from 10 large referral centers for ILDs in two different countries (Greece and Denmark), including Department of Internal and Respiratory Medicine, University Hospital of Patras, 1st and 2nd Academic Department of Respiratory Medicine, “SOTIRIA” and “ATTIKON” General Hospital, National and Kapodistrian University of Athens, 5th and 7th Department of Respiratory Medicine, “SOTIRIA” General Hospital for Thoracic Diseases, Department of Respiratory Medicine “Corfu General Hospital”, Department of Respiratory Medicine, University General Hospital of Alexandroupolis, Department of Respiratory Medicine, Medical School, University of Ioannina, Pulmonary Department of Aristotle University of Thessaloniki, “Papanikolaou” General Hospital, and Center for Rare Lung Diseases, Department of Respiratory Diseases and Allergy, Aarhus University Hospital.

The study was approved by the Institutional Review Board and the Local Ethics Committee (Protocol Number: 5792/6-3-19). Diagnosis of HP was based on current 2020 ATS/JRS/ALAT guidelines and following multidisciplinary discussion [1]. All patients recruited for the study and included in the analysis presented with imaging features typical of, or at least compatible with, HP in the context of a confident pre-test clinical probability. Patients who did not meet the current diagnostic criteria for HP or with indeterminate-for-HP/unclassified disease patterns, patients with incomplete clinical data (e.g., pre-treatment CBC), and individuals who were not treatment-naïve were excluded from our analysis.

We collected parameters of CBC, including monocytes, eosinophils, neutrophils, and lymphocytes, as well as blood cell ratios, including the eosinophil–monocyte ratio (EMR), eosinophil–lymphocyte ratio (ELR), and neutrophil–lymphocyte ratio (NLR), prior to treatment initiation. Demographics, such as age, gender, smoking status, history of exposure, and survival data were also collected. We documented comorbidities, including arterial hypertension, pulmonary hypertension, thyroid disorders, gastroesophageal reflux disease, and diabetes mellitus, based on physicians’ assessments and patients’ medical records. We also recorded functional indices, such as Forced Vital Capacity (% predicted) and Diffusing Capacity of the Lungs for carbon monoxide in order to calculate each patient’s baseline Gender–Age–Physiology (GAP) score. The GAP index is a validated clinical tool originally developed to predict mortality in IPF, incorporating sex, age, and lung function measures into a composite score. Although primarily designed for IPF, it has also been applied to other fibrotic ILDs, including HP, to aid in risk stratification and prognosis [13,14,15].

### 2.1. Outcome Measures

The endpoints of this study were to investigate the prognostic performance of peripheral blood cell parameters (1) in patients with HP and (2) between patients with non-fibrotic and fibrotic HP in terms of mortality.

### 2.2. Statistical Analysis

Kaplan–Meier survival analysis was applied to investigate differences in survival between groups. Survival analysis using Cox regression adjusted to GAP scores was also performed. Patients were split into two groups (high and low) following the use of receiver operating characteristic (ROC) curves, which identified the optimal thresholds for eosinophil, monocyte, neutrophil, and lymphocyte counts, as well as for blood cell ratios, including EMR, ELR, and NLR, in terms of mortality prediction. Differences in parameters of CBC between patients with non-fibrotic and fibrotic HP were investigated using Mann–Whitney or *t*-tests based on the absence or the presence of normality. Continuous variables are presented as means ± standard deviation (SD) if data followed a normal distribution or medians [95% confidence interval (CI)] if not. The results are presented in tables and figures.

## 3. Results

### 3.1. Baseline Characteristics

We included 129 patients who were diagnosed with HP following multidisciplinary discussion and based on the current 2020 ATS/ERS guidelines [1]. The majority of patients were diagnosed with fibrotic HP (*n* = 85, 65.9%), while 44 patients were diagnosed with non-fibrotic HP (34.1%), as assessed by high-resolution computed tomography patterns. The median age (95% CI) was 68.0 (65.0 to 69.0) years. The median eosinophil count (95% CI) at baseline was 210 (200 to 251) cells/μL. The mean monocyte count (±SD) at baseline was 562 ± 224 cells/μL. The most common comorbidities were arterial hypertension (*n* = 71, 55%) and dyslipidemia (*n* = 56, 43.4%). Detailed baseline characteristics are presented in Table 1. All patients (*n* = 129, 100%) were treatment-naïve at the time point of inclusion. The treatment regimen following baseline evaluation is presented in Table 2. The most commonly prescribed compounds following baseline evaluation were oral corticosteroids (*n* = 97, 75.2%), inhaled corticosteroids (*n* = 30, 23.3%), and mycophenolic acid (*n* = 26, 20.2%).

### 3.2. Increased Number of Eosinophils and Monocytes Is Associated with Worse Survival

**Figure 1:**
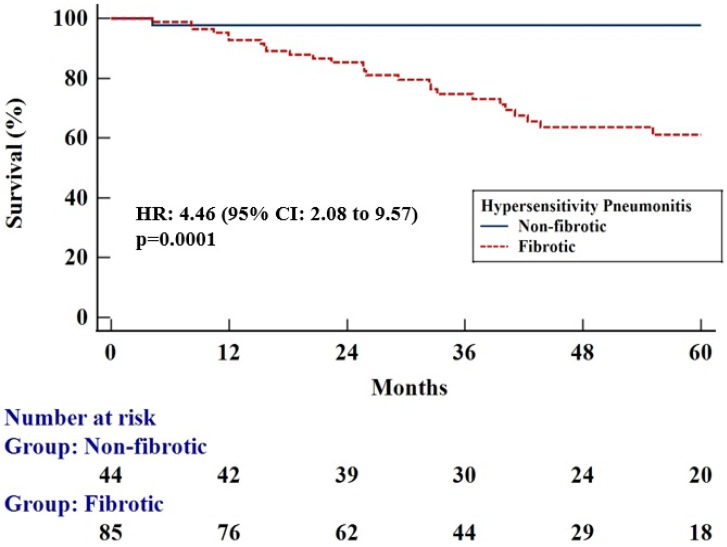
Kaplan–Meier curve showing survival difference between patients with fibrotic (*n* = 85) and non-fibrotic (*n* = 44) HP [HR: 4.46 (95% CI: 2.08 to 9.57), *p* = 0.0001].

**Figure 2:**
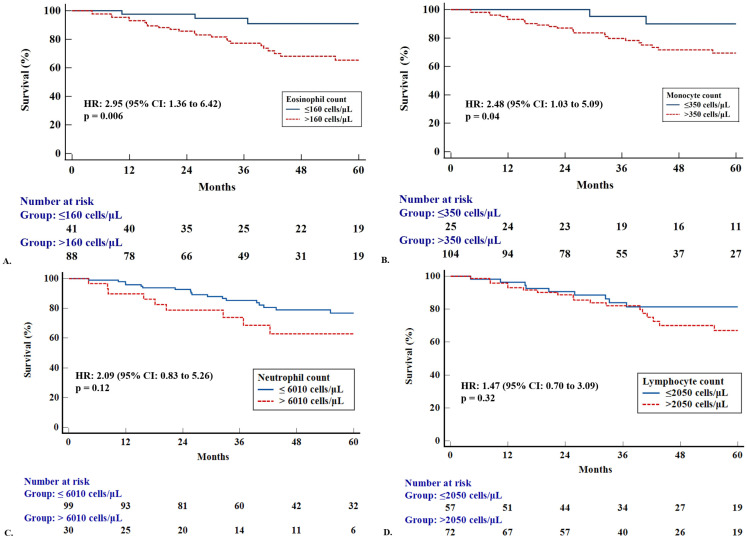
Kaplan–Meier curves showing survival differences according to complete blood count cell parameters. (**A**) Patients with high (*n* = 88) versus low (*n* = 41) eosinophil count with cut-off point > 160 cells/μL [HR: 2.95 (95% CI: 1.36 to 6.42), *p* = 0.006]. (**B**) Patients with high (*n* = 104) versus low (*n* = 25) monocyte count with cut-off point of 350 cells/μL [HR: 2.48 (95% CI: 1.03 to 5.09), *p* = 0.04]. (**C**) Patients with high (*n* = 30) versus low (*n* = 99) neutrophil count with cut-off point of 6010 cells/μL [HR: 2.09 (95% CI: 0.83 to 5.26), *p* = 0.12]. (**D**) Patients with high (*n* = 72) versus low (*n* = 57) lymphocyte count with cut-off point of 2050 cells/μL [HR: 1.47 (95% CI: 0.7 to 3.09), *p* = 0.32].

Kaplan–Meier analysis showed that patients with fibrotic HP had an increased mortality risk compared to patients with non-fibrotic HP [hazard ratio (HR): 4.46 (95% CI: 2.08 to 9.57), *p* = 0.0001], (Figure 1). Subsequently, we tested whether parameters of complete blood count could predict outcomes in the whole HP population. ROC curve analysis identified the value “>160 cells/μL” as the optimal cut-off threshold for the eosinophil count to discriminate patients into mortality risk groups [area under the curve (AUC): 0.61]. Patients with HP and an eosinophil count > 160 cells/μL exhibited increased mortality risk compared to patients with HP and an eosinophil count ≤ 160 cells/μL [Kaplan–Meier, HR: 2.95 (95% CI: 1.36 to 6.42), *p* = 0.006] (Figure 2A). Patients with HP and an eosinophil count > 160 cells/μL exhibited increased mortality risk even following Cox-regression adjusted to GAP scores [HR: 3.73 (95% CI: 1.12 to 12.37), *p* = 0.03]. ROC curve analysis identified the threshold of “350 cells/μL” as the optimal cut-off value for monocyte counts to discriminate patients into mortality risk groups (AUC: 0.52). Patients with HP and a monocyte count > 350 cells/μL exhibited worse survival compared to patients with a monocyte count < 350 cells/μL [Kaplan–Meier, HR: 2.48 (95% CI: 1.03 to 5.09), *p* = 0.04] (Figure 2B). Cox regression adjusted to GAP scores also showed that patients with HP and a monocyte count > 350 cells/μL displayed increased mortality [HR: 4.25 (95% CI: 1.00 to 17.97), *p* = 0.04]. ROC curve analysis identified the value “>6010 cells/μL” as the optimal cut-off threshold for neutrophil counts to discriminate patients into mortality risk groups (AUC: 0.52). Patients with HP and a neutrophil count > 6010 cells/μL did not exhibit a statistically significant increased mortality risk compared to patients with HP and a neutrophil count ≤ 6010 cells/μL [Kaplan–Meier, HR: 2.09 (95% CI: 0.83 to 5.26), *p* = 0.12] (Figure 2C). Finally, ROC curve analysis identified the value “>2050 cells/μL” as the optimal cut-off threshold for lymphocyte counts to discriminate patients into mortality risk groups (AUC: 0.52). Patients with HP and a lymphocyte count > 2050 cells/μL did not exhibit a statistically significant increased mortality risk compared to HP and a lymphocyte count ≤ 2050 cells/μL [Kaplan–Meier, HR:1.47 (95% CI: 0.70 to 3.09), *p* = 0.32] (Figure 2D). Cox regression adjusted to GAP scores was not performed for neutrophils and lymphocytes.

### 3.3. Increased ELR Is Associated with Worse Survival

**Figure 3:**
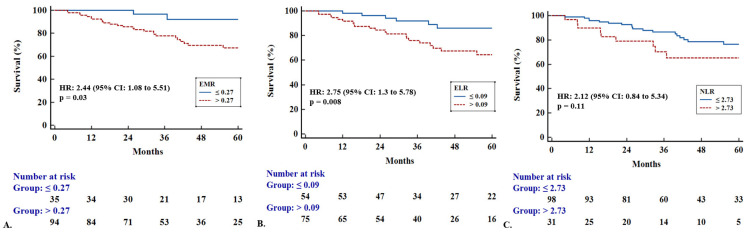
Kaplan–Meier curves showing survival differences according to blood cell ratios (**A**). Patients with high (*n* = 94) versus low (*n* = 35) eosinophil–monocyte ratio (EMR) with cut-off point of 0.27 [HR: 2.44 (95% CI: 1.08 to 5.51), *p* = 0.03]. (**B**). Patients with high (*n* = 75) versus low (*n* = 54) eosinophil–lymphocyte ratio (ELR) with cut-off point of 0.09 [HR: 2.75 (95% CI: 1.3 to 5.78), *p* = 0.008]. (**C**). Patients with high (*n* = 31) versus low (*n* = 98) neutrophil–lymphocyte ratio (NLR) with cut-off point of 2.73 [HR: 2.12 (95% CI: 0.84 to 5.34), *p* = 0.11].

Furthermore, we tested whether blood cell ratios, including EMR, ELR, and NLR, could predict outcomes in the whole HP population. ROC curve analysis identified the value “0.27” as the optimal cut-off threshold for EMR to discriminate patients into mortality risk groups [AUC: 0.59]. Patients with HP and EMR > 0.27 exhibited increased mortality risk compared to patients with HP and EMR ≤ 0.27 [Kaplan–Meier, HR: 2.44 (95% CI: 1.08 to 5.51), *p* = 0.03] (Figure 3A). Moreover, ROC curve analysis identified the value “0.09” as the optimal cut-off threshold for ELR [AUC: 0.64]. Patients with HP and ELR > 0.09 had a higher risk of mortality compared to patients with HP and ELR ≤ 0.09 [Kaplan–Meier, HR: 2.75 (95% CI: 1.3 to 5.78), *p* = 0.008] (Figure 3B). Finally, ROC curve analysis identified the value “2.73” as the optimal cut-off threshold for NLR [AUC: 0.53]. Patients with HP and NLR > 2.73 did not exhibit statistically significant increased mortality risk compared to patients with HP and NLR ≤ 2.73 [Kaplan–Meier, HR: 2.12 (95% CI: 0.84 to 5.34), *p* = 0.11] (Figure 3C). Survival analysis using Cox regression adjusted to GAP scores was also performed. Patients with HP and ELR > 0.09 exhibited increased mortality risk even following Cox regression adjusted to GAP scores [HR: 3.05 (95% CI: 1.23 to 7.6), *p* = 0.02], while patients with HP and EMR > 0.27 did not [HR: 2.63 (95% CI: 0.79 to 8.79), *p* = 0.11]. Cox regression adjusted to GAP scores was not performed for NLR.

### 3.4. Increased Number of Eosinophils Differentiates Fibrotic from Non-Fibrotic HP

**Figure 4:**
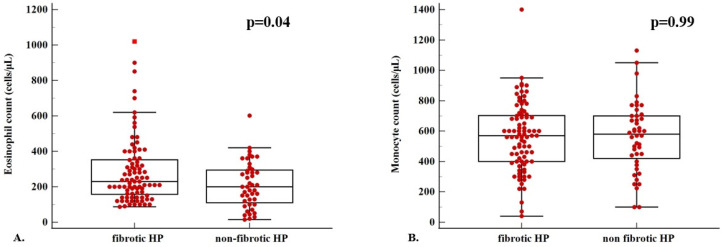
(**A**) Eosinophil count (median, 95% CI) in patients with fibrotic (*n* = 85) vs. non-fibrotic (*n* = 44) HP [230 (95% CI: 200 to 280) vs. 200 (95% CI: 150 to 270), *p* = 0.04, Mann–Whitney test]. (**B**) Monocyte count (mean ± SD) in patients with fibrotic (*n* = 85) vs. non-fibrotic (*n* = 44) HP (561 ± 224 vs. 562 ± 228, *p* = 0.99, independent samples *t*-test).

Patients with fibrotic HP had a higher baseline eosinophil count (cells/μL) compared to patients with non-fibrotic HP [230 (95% CI: 200 to 280) vs. 200 (95% CI: 150 to 270), *p* = 0.04] (Figure 4A). There was no significant difference in terms of monocyte count (cells/μL) between patients with fibrotic and non-fibrotic HP (561 ± 224 vs. 562 ± 228, *p* = 0.99) (Figure 4B).

## 4. Discussion

To our knowledge, this study is the first to demonstrate the prognostic usefulness of clinically accessible biomarkers from peripheral blood cell counts in patients with HP. In particular, both eosinophil and monocyte counts, as well as the inflammatory ratio ELR, could predict mortality in univariate and multivariate analysis in a large cohort of highly characterized patients with fibrotic and non-fibrotic forms of HP. Importantly, our analysis included treatment-naïve patients, which allowed us to capture the unmodified immune profile of the disease. This is a key strength of our study and may help explain the discrepancy with previous reports, where patients were already receiving treatment, potentially altering peripheral cell counts and their prognostic significance.

Our study exhibited a number of major attributes, further analyzed below:

(1) The finding that eosinophils might be prognostic biomarkers in HP is the most novel attribute of this work. Despite the lack of prognostic data for eosinophils in HP, several studies have shown that eosinophils are often present in lung tissue biopsies of patients with HP [2,5]. In non-fibrotic forms of HP, histopathological evidence has demonstrated peribronchiolar inflammatory infiltrates with lymphocytes, plasma cells, and occasional eosinophils with associated small, non-necrotizing, poorly formed granulomas [16]. While HP was traditionally considered to be associated with a Th1-mediated hypersensitivity response to antigens in the environment, there seem to be relevant features to the Th2-response [16]. In particular, reports have shown higher sputum eosinophils and upregulated T2-high inflammatory markers [interleukin(IL)-4, IL-5] following specific inhalation challenge in patients with bird-related HP versus those with HP associated with fungi [8]. Subsequent investigations demonstrated a Th2-mediated lung injury in patients with chronic HP with an increased BAL fluid CD4/CD8 ratio and upregulated levels of T-cell-induced IL-4, further supporting the premise of a Th2-mediated inflammatory response [17]. Therefore, a Th1-dominant environment—marked by cytokines such as interferon-γ, tumor necrosis factor-α, IL-2, and IL-12—appears to primarily drive early immune responses; however, a shift toward a Th2 profile, involving IL-4 and IL-13, may contribute to persistent inflammation and the development of fibrosis in later stages of the disease [2,17,18,19]. Moreover, our finding that increased ELR is associated with worse prognosis in patients with HP further strengthens the role of eosinophilic inflammation in the pathophysiology of HP. ELR has shown usefulness as a prognostic marker in various respiratory diseases, such as asthma and chronic obstructive pulmonary disease, serving as a reliable indicator of eosinophilic airway inflammation [20,21]. Other blood cell ratios may also serve as markers of the underlying pathophysiological states of pulmonary diseases, for example, NLR in community-acquired pneumonia, which reflects the predominantly neutrophilic nature of the inflammatory response [22,23,24]. Overall, the latter evidence raises the hypothesis of a distinct eosinophil/Th2-driven endotype in some patients with HP, possibly sharing immunologic features with asthma or eosinophilic disorders, and suggests that a subset of patients could potentially benefit from anti-eosinophilic or other biologic agents. The latter hypothesis remains to be proven.
(2) Our finding that an increased monocyte count is a negative prognostic marker in HP validates previous studies showing the predictive role of monocyte counts in IPF and other ILDs, particularly fibrotic forms [6,7,25,26,27,28,29,30]. Currently, there is compelling evidence supporting the major role of monocytes in the pathogenesis of HP. A recent, elegant study using single-cell RNA sequencing in peripheral blood mononuclear cells and BAL from patients with fibrotic HP provided important insights in this direction [31]. In this work, patients with fibrotic HP exhibited higher numbers of S100A^hi^ and CCL3/CCL4^hi^ classical monocytes compared to healthy individuals. These monocytes seemed to have increased pro-inflammatory transcription factor activities and developed into SPP1^hi^ pro-fibrotic macrophages based on the trajectory analysis conducted. It is noteworthy that SPP1^hi^ pro-fibrotic macrophages have been described in IPF [32]. This implies shared immune fibrotic responses in IPF and fibrotic HP. However, some parts of the pathogenesis differ; for example, GZM^hi^ cytotoxic T cells are present only in fibrotic HP, while fibrogenesis is associated with particular clusters of monocytes and macrophages in both diseases [31,33]. In the context of IPF, we and other investigators have shown that elevated peripheral blood monocyte counts are strongly associated with worse clinical outcomes; unfortunately, prognostic cut-off thresholds are highly variable, thus limiting, so far, their clinical applicability [6,26]. In particular, retrospective pooled analysis from three large databases and the analysis of the Australian IPF registry have identified an increased monocyte count as a potential negative prognostic marker in IPF [6,34]. A pooled retrospective analysis of data from ASCEND, CAPACITY, and INSPIRE demonstrated that patients with IPF and a monocyte count in the range of 600–950 cells/μL or ≥950 cells/μL had a higher 1-year risk of disease progression, all-cause hospitalization, and all-cause mortality compared to patients with a monocyte count of <600 cells/μL [7]. In the context of HP, contrary to our findings, a recent study showed that monocyte counts did not predict outcomes in fibrotic HP [27]. This might be due to the fact that a pure fibrotic HP population is not necessarily treatment-naïve, particularly with regard to systemic corticosteroids and other immunomodulatory agents, in contrast with the population of our study, which is unique with respect to baseline data prior to treatment initiation [35,36]. Thus, the baseline, treatment-naïve monocyte count may serve as a potential biomarker of mortality in patients with HP.
(3) Finally, our study represents one of the first efforts to identify clinically applicable biomarkers of mortality in patients with HP. So far, the only available biomarker widely used in clinical practice is BAL lymphocytosis, with more diagnostic than prognostic granularity [18,37]. CBC parameters and blood cell ratios hold promise as practical prognostic biomarkers for HP, given their accessibility, cost-effectiveness, and suitability for serial measurements. Other suggested biomarkers such as chitinase 3-like 1, Krebs Von Den Lungen-6, Club-Cell Protein 16, and lipid mediators are neither disease-specific nor clinically applicable due to both technical and financial issues [18]. As more data emerge and cut-offs are refined through larger, prospective cohorts, a prognostic scoring system integrating CBC parameters—potentially along with laboratory, clinical, and imaging markers—could be developed to enhance risk stratification and support clinical decision-making.

Despite the relative enthusiasm arising from the above data and the important attributes presented, our study has some limitations that should be treated cautiously. First, this study has the inherent weaknesses of a retrospective study. However, the nature of this study let us present the long-term outcomes of patients with HP. Second, our sample size is moderate, and thus larger studies are greatly needed to extract rigid conclusions. Yet, the sample size was adequate to present the importance of clinically applicable biomarkers in HP. Thirdly, our study was not designed to provide mechanistic insights, which could be an interesting topic for future research. Moreover, we analyzed all-cause mortality and not solely respiratory-related deaths, did not apply adjustments for multiple comparisons, and did not account for confounding factors, such as prescribed treatment or fibrotic status, in relation to mortality outcomes. Future studies should address these factors to refine and strengthen prognostic models. Finally, given the inconsistency and limited availability of established prognostic cut-offs, we utilized ROC curve analysis to identify optimal thresholds for mortality prediction. The cut-offs determined in our analysis were significantly associated with survival; however, their predictive accuracy remains moderate, emphasizing the need for future prospective validation in larger cohorts before they can be applied in clinical practice and before rigid conclusions can be drawn.

## 5. Conclusions

In conclusion, this study demonstrated that eosinophil count and monocyte count could be prognostic biomarkers in patients with HP. The implementation of clinically applicable biomarkers is extremely important for ILDs, given the paucity of such markers in current clinical practice. Larger studies investigating the role of CBC in patients with HP are sorely needed. The latter could identify potential therapeutic targets, particularly for fibrotic forms, whichshare a similar dismal prognosis with IPF, as a subgroup of patients may benefit from anti-eosinophilic agents, which are currently the first line treatment applied to patients with severe asthma and other eosinophilic lung disorders.

## Figures and Tables

**Table 1:** Baseline characteristics of patients included in the study.

Characteristics	(*N*, %)
Total number of patients	129
Total number of deaths	29
Age (median, 95% CI)	68.0 (65.0 to 69.0)
Male/Female	75 (58.1%)/54 (41.9%)
Smoking history: Current/Ever/Never	14 (10.9%)/63 (48.8%)/52 (40.3%)
Fibrotic/Non-fibrotic HP	85 (65.9%)/44 (34.1%)
Exposure/No Exposure	112 (86.8%)/17 (13.2%)
Eosinophil count baseline (median, 95% CI)	210 (200 to 251) cells/μL
Monocyte count baseline (mean, ±SD)	562 ± 224 cells/μL
FVC baseline %predicted (mean, ±SD)	76.7 ± 20.1%
Arterial hypertension	71 (55%)
Dyslipidemia	56 (43.4%)
GERD	53 (41.1%)
Diabetes mellitus	26 (20.2%)
Pulmonary hypertension	25 (19.4%)
Hypothyroidism	19 (14.7%)
Finger clubbing	50 (38.8%)
LTOT	34 (26.4%)

Abbreviations: CI, confidence interval; FVC, forced vital capacity; HP, hypersensitivity pneumonitis; GERD, gastroesophageal reflux disease; LTOT, long-term oxygen therapy; SD, standard deviation.

**Table 2:** Prescribed treatment for hypersensitivity pneumonitis after inclusion in the study.

Treatment	(N, %)
Total number of patients	129
Oral corticosteroids	97 (75.2%)
Inhaled corticosteroids	30 (23.3%)
Mycophenolic acid	26 (20.2%)
Pirfenidone	16 (12.4%)
Nintedanib	30 (23.3%)
Azathioprine	3 (2.3%)
Rituximab	1 (0.8%)
No treatment—exposure avoidance	10 (7.8%)

## Data Availability

The original contributions presented in this study are included in the article. Further inquiries can be directed to the corresponding author.

## Abbreviations

The following abbreviations are used in this manuscript:

HP	Hypersensitivity pneumonitis
HR	Hazard ratio
IPF	Idiopathic pulmonary fibrosis
CBC	Complete blood count
ILD	Interstitial lung disease
CI	Confidence interval
FVC	Forced vital capacity
GAP	Gender–Age–Physiology
ROC	Receiver operating characteristic
AUC	Area under the curve
GERD	Gastroesophageal reflux disease
SD	Standard deviation
LTOT	Long-term oxygen therapy
